# m5CPred-SVM: a novel method for predicting m5C sites of RNA

**DOI:** 10.1186/s12859-020-03828-4

**Published:** 2020-10-30

**Authors:** Xiao Chen, Yi Xiong, Yinbo Liu, Yuqing Chen, Shoudong Bi, Xiaolei Zhu

**Affiliations:** 1 School of Sciences, Anhui Agricultural University, Hefei, 230036 Anhui China; 2 School of Life Sciences and Biotechnology, Shanghai Jiao Tong University, Shanghai, 200240 China

**Keywords:** 5-Methylcytosine sites, Support vector machine, Computational predictor, Position specific propensity, Web server

## Abstract

**Background:**

As one of the most common post-transcriptional modifications (PTCM) in RNA, 5-cytosine-methylation plays important roles in many biological functions such as RNA metabolism and cell fate decision. Through accurate identification of 5-methylcytosine (m5C) sites on RNA, researchers can better understand the exact role of 5-cytosine-methylation in these biological functions. In recent years, computational methods of predicting m5C sites have attracted lots of interests because of its efficiency and low-cost. However, both the accuracy and efficiency of these methods are not satisfactory yet and need further improvement.

**Results:**

In this work, we have developed a new computational method, m5CPred-SVM, to identify m5C sites in three species, *H. sapiens*, *M. musculus* and *A. thaliana*. To build this model, we first collected benchmark datasets following three recently published methods. Then, six types of sequence-based features were generated based on RNA segments and the sequential forward feature selection strategy was used to obtain the optimal feature subset. After that, the performance of models based on different learning algorithms were compared, and the model based on the support vector machine provided the highest prediction accuracy. Finally, our proposed method, m5CPred-SVM was compared with several existing methods, and the result showed that m5CPred-SVM offered substantially higher prediction accuracy than previously published methods. It is expected that our method, m5CPred-SVM, can become a useful tool for accurate identification of m5C sites.

**Conclusion:**

In this study, by introducing position-specific propensity related features, we built a new model, m5CPred-SVM, to predict RNA m5C sites of three different species. The result shows that our model outperformed the existing state-of-art models. Our model is available for users through a web server at https://zhulab.ahu.edu.cn/m5CPred-SVM.

## Background

Over 170 chemical modifications have been discovered in both coding and non-coding RNAs to date [1–3]. 5-cytosine-methylation is one of the most common post-transcriptional modifications (PTCM) and has been found in almost all types of RNA [4, 5]. This modification can regulate nuclear mRNA output and RNA variable splicing, increase RNA stability, regulate protein translation and RNA–protein interaction, and maintain the normal structure of RNA [6–14]. Under the catalysis of RNA methyltransferase, methylation occurs on the carbon atom in the fifth position of a cytosine to generate 5-methylcytosine (m5C). Therefore, accurate identification of m5C sites in RNA is of great importance for understanding the mechanism and function of this modification.

Both experimental and computational methods have been developed to determine and predict m5C sites in RNA. Experimental methods such as bisulfite sequencing [5, 12], m5C-RIP [15], Aza-IP [16], mi-CLIP [17] and RBS-seq [18] have been somewhat successful in identification of m5C sites in RNAs of different species. However, these experimental methods are time-consuming and expensive, and they are not able to keep pace with the explosive increase of RNA sequences revealed by the rapid development of sequencing technology. Instead, computational methods can be able to provide a faster and more cost-effective way for m5C site identification.

So far, eight computational methods for predicting m5C site have been reported, which were summarized in Table 1 according to the datasets, algorithms, webservers, evaluation strategies and features employed. Feng et al. [19] built their model using a support vector machine based on PseKNC features extracted from RNA segments, and a balanced dataset with 120 m5C sites and 120 non-m5C sites was used to build this model. In addition, nine other datasets with 120 non-m5C sites were randomly selected to demonstrate their model is not sensitive to the selection of non-m5C sites. Later, Qiu et al. [20] have developed a model called iRNAm5C-PseDNC to predict m5C sites by using random forests. Differently, this model was built on an imbalanced and redundant dataset with 475 m5C sites and 1425 non-m5C sites. Then, by using ensemble learning methods, Zhang et al. [21] have developed a model called m5C-HPCR. A new heuristic algorithm was introduced to reduce the number of physical and chemical properties of nucleotides. The m5C-HPCR was benchmarked on both Feng et al.’s dataset and Qiu et al.’s dataset. Sabooh et al. [22] have developed a model by fusing composite encoding features including Di-Nucleotide Composition (DNC), Tri-Nucleotide Composition (TNC) and Tetra- Nucleotide Composition (TetraNC). The same dataset as that of Feng et al. [19] and Zhang et al. [21] was again used to build this model by using SVM. Recently, Fang et al. [23] compared the balanced dataset used in Feng et al.’s work and the imbalanced dataset used in Qiu et al.’s work, and developed a model named RNAm5CPred to predict m5C sites of *H. sapiens*. The model was built by SVM and an independent test set was used to evaluate different methods. A new predictor (PEA-m5C) developed by Song et al. [24] mainly focused on predicting m5C sites in *A. thaliana.* The model was built by using random forests on an imbalanced dataset but was tested on three balanced independent datasets. Li et al. [25] had collected data from GEO database and developed a web server RNAm5Cfinder based on random forest algorithm, which can be used to predict m5C sites in eight kinds of cells or tissues of mouse and human. All the m5C sites recorded in three GEO records and all other non-m5C sites in the genomes were collected to train their models, however, the redundancy of the datasets was not well dealt with. More recently, Lv et al. [26] developed a server called iRNA-m5C to predict m5C sites of four types of species. Their models are built with random forests with features of PseKNC, MNBE (mono-nucleotide binary encoding), KNFC(K-tuple nucleotide frequency component) and NV (natural vector).

**Table 1:** Summarization of the existing methods for predicting m5C sites of RNA

Methods	Datasets^a^	Algorithms	Webserver availability	Evaluation strategy	Features	Species
iRNA-m5C [26]	120 m5C + 120 non-m5C 97 m5C + 97 non-m5C 6289 m5C + 6289 non-m5C 211 m5C + 211 non-m5C	RF	Yes	(1) Jackknife test (2) independent test	PseKNC MNBE KNFC NV	*H. sapiens* *M.musculus* *A. thaliana* *S.cerevisiae*
RNAm5Cfinder [25]	All m5C sites recorded in GSE90963 GSE93749 GSE83432	RF	Yes	(1) Fivefold cross validation (2) Independent test	MNBE	*H. sapiens* *M. musculus*
PEA-m5C [24]	DatasetCV (1196:11960) DatasetHT (100:100) DatasetT1 (79:79) DatasetT2 (73:73)	RF	Yes	(1) Tenfold cross validation (2) Independent test	PseDNC KNFC MNBE	*A. thaliana*
RNAm5CPred [23]	Met935 (127:808) Met240 (120:120) Met1900 (475:1425) Test1157 (157:1000)	SVM	Yes	(1) Jackknife test (2) Tenfold cross validation (3) Independent test	KNF KSNPF PseDNC	*H. sapiens*
pM5CS-Comp-mRMR [22]	120 m5C and 120 non-m5C	SVM	No	Jackknife test	DNC, TNC, Tetra-NC	*H. sapiens*
M5C-HPCR [21]	Met1320(120:1200)^b^ Met1900 (475:1425)	Ensemble of SVM	No	Jackknife test	PseDNC	*H. sapiens*
iRNAm5C-PseDNC [20]	Met1900 (475:1425)	RF	Yes	Jackknife test	PseDNC	*H. sapiens*
m5C-PseDNC [19]	Met1320(120:1200)^b^	SVM	No	Jackknife test	PseDNC	*H. sapiens*

^a^The numbers in the parentheses are the ratios between m5C and non-m5C sites of that dataset

^b^Although the ratio between m5C and non-m5C sites is 120:1320, but the final model is based on a balanced dataset with 120 m5C and 120 non-m5C sites

Although these reported methods performed well in the recognition of m5C sites in animal and plant RNA sequences, it is possible that the performance can be improved by introducing position specific related features such as position specific nucleotide propensity (PSNP), position specific dinucleotide propensity (PSDP). The effectiveness of these features has been proved in previous works [27, 28] for predicting m6A of RNA, however, the use of these features to predict m5C sites has not been explored in these methods mentioned above. It is expected that the performance of computational methods can be further improved by mining position specific related features and composition related features.

In this study, we have developed a new method, m5CPred-SVM, to predict m5C sites in RNA sequences of three different species, *H. sapiens*, *M. musculus* and *A. thaliana*. First, we generated six kinds of features based on RNA sequences, namely k-nucleotide frequency (KNF), pseudo dinucleotide composition (pseDNC), K-spaced nucleotide pair frequency (KSNPF), position-specific nucleotide propensity (PSNP), K-spaced position-specific dinucleotide propensity (KSPSDP) and Chemical Property with Density (CPD). Then, the sequential forward feature selection strategy was used to select a compact feature subset from these six kinds of features. Based on this selected feature subset, our method was built using a support vector machine (SVM). At last, the performance of our method was compared with several existing methods. The results showed that our method can offer substantially better performance than these existing methods on the independent test sets.

## Results

### Performance of each type of feature

**Fig. 1:**
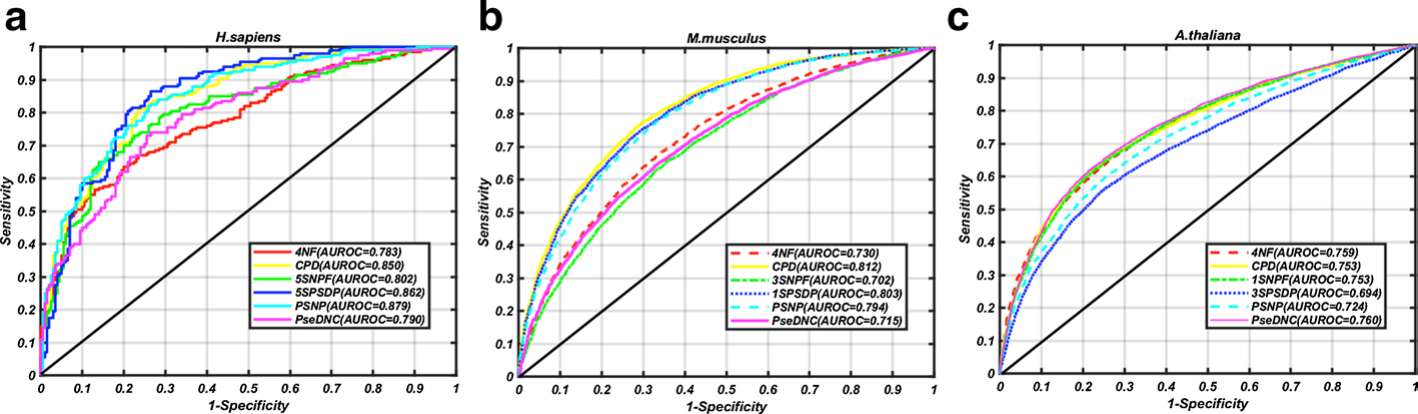
The ROC curves that show the performances of the six type of features for *H. sapiens*, *M. musculus* and *A. thaliana*, respectively. **a** *H. sapiens*; **b** *M. musculus*; **c** A*. thaliana*

By using SVM over the ten folds cross-validation, we have evaluated the performances of the six types of extracted features for the three species, namely *H. sapiens*, *M. musculus* and *A. thaliana*. As shown in Table 2, PSNP, KSPSDP, CPD are the three features showing the best performances among the six types of features for *H. sapiens.* The cross-validation AUROCs for these three features are 0.879, 0.862 and 0.850, respectively. Table 3 shows that CPD, KSPSDP and PSNP are the three features providing the best performances for *M. musculus*. The cross-validation AUROCs are 0.812, 0.803 and 0.794 for these three features, respectively. As for *A. thaliana*, Table 4 shows that the top three models with the best performances were based on PseDNC, 4NF and CPD, and the corresponding cross-validation AUROCs are 0.760, 0.759 and 0.753. The ROC curves of the six types of features for *H. sapiens*, *M. musculus* and *A. thaliana* are shown in Fig. 1.

**Table 2:** The results of feature selection for *H. sapiens*

Feature subset	KS	BC	Sn (%)	Sp (%)	Pre (%)	Acc (%)	Mcc	F1score	AUC
PSNP	1	32	81.5	81.0	81.1	81.3	0.625	0.813	0.879
5SPSDP	0.25	8	82.5	77.5	78.6	80.0	0.601	0.805	0.862
CPD	8	0.25	81.0	76.5	77.5	78.8	0.576	0.792	0.850
5SNPF	0.25	4	73.5	79.5	78.2	76.5	0.531	0.758	0.802
PseDNC	1	4096	74.0	73.0	73.3	73.5	0.470	0.736	0.790
4NF	0.125	0.5	55.5	88.5	82.8	72.0	0.466	0.665	0.783
PSNP + 4NF	1	16	83.5	80.0	80.7	81.8	0.635	0.821	0.893
PSNP + 5SNPF	1	32	81.0	80.5	80.6	80.8	0.615	0.808	0.882
PSNP + 5SPSDP	2	32	82.5	81.0	81.3	81.8	0.635	0.819	0.885
PSNP + PseDNC	0.5	0.25	87.5	70.5	74.8	79.0	0.589	0.806	0.854
PSNP + CPD	8	0.25	82.0	75.5	77.0	78.8	0.576	0.794	0.850
PSNP + 4NF + 5SNPF	1	16	85.5	79.5	80.7	82.5	0.651	0.830	0.897
PSNP + 4NF + 5SPSDP	1	8	82.5	82.5	82.5	82.5	0.650	0.825	0.895
PSNP + 4NF + CPD	8	0.25	82.0	75.5	77.0	78.8	0.576	0.794	0.850
PSNP + 4NF + PseDNC	1	16	80.5	78.5	78.9	79.5	0.590	0.797	0.873
PSNP + 4NF + 5SNPF + 5SPSDP	1	8	81.5	80.5	80.7	81.0	0.620	0.811	0.896
PSNP + 4NF + 5SNPF + CPD	64	16	84.0	73.0	75.7	78.5	0.573	0.796	0.854
PSNP + 4NF + 5SNPF + PseDNC	1	16	85.5	80.0	81.0	82.8	0.656	0.832	0.899
PSNP + 4NF + 5SNPF + PseDNC + CPD	64	16	84.0	73.0	75.7	78.5	0.573	0.796	0.854
PSNP + 4NF + 5SNPF + PseDNC + 5SPSDP	1	8	81.5	81.5	81.5	81.5	0.630	0.815	0.897

**Table 3:** The results of feature selection for *M. musculus*

Feature subset	KS	BC	Sn (%)	Sp (%)	Pre (%)	Acc (%)	Mcc	F1score	AUC
CPD	4	1	74.0	73.1	73.3	73.6	0.461	0.737	0.812
1SPSDP	16	8192	73.0	72.6	72.7	72.8	0.456	0.728	0.803
PSNP	8	8192	75.2	69.1	70.9	72.2	0.444	0.730	0.794
4NF	0.125	0.5	68.0	66.1	66.8	67.1	0.341	0.674	0.730
PseDNC	0.5	256	65.1	66.2	65.8	65.7	0.313	0.655	0.715
1SNPF	1	8	65.5	64.2	64.7	64.9	0.298	0.652	0.702
CPD + 1SPSDP	4	1	74.1	73.1	73.3	73.6	0.472	0.737	0.813
CPD + PSNP	4	1	74.2	73.0	73.3	73.6	0.472	0.738	0.813
CPD + 4NF	32	256	75.1	72.7	73.3	73.9	0.478	0.742	0.815
CPD + PseDNC	64	4096	75.4	72.4	73.2	73.9	0.478	0.743	0.813
CPD + 1SNPF	8	2	74.8	72.3	73.0	73.6	0.471	0.739	0.811
CPD + 4NF + 1SNPF	32	256	75.3	72.7	73.4	74.0	0.480	0.743	0.816
CPD + 4NF + PSNP	32	256	75.2	72.7	73.3	73.9	0.479	0.742	0.815
CPD + 4NF + 1SPSDP	64	4096	75.7	72.8	73.6	74.3	0.486	0.746	0.822
CPD + 4NF + PseDNC	32	256	75.4	72.7	73.4	74.0	0.48	0.744	0.816
CPD + 4NF + 1SPSDP + 1SNPF	64	2048	76.0	72.9	73.8	74.5	0.490	0.749	0.822
CPD + 4NF + 1SPSDP + PSNP	64	4096	75.7	72.8	73.6	74.2	0.485	0.746	0.822
CPD + 4NF + 1SPSDP + PseDNC	64	4096	75.7	72.8	73.6	74.2	0.485	0.746	0.822

**Table 4:** The results of feature selection for *A. thaliana*

Feature subset	KS	BC	Sn (%)	Sp (%)	Pre (%)	Acc (%)	Mcc	F1score	AUC
PseDNC	0.125	0.25	59.4	80.6	75.4	70.0	0.410	0.665	0.760
4NF	0.125	1	62.3	76.3	72.4	69.3	0.389	0.670	0.759
CPD	16	16	61.1	78.4	73.9	69.8	0.401	0.669	0.753
1SNPF	0.25	0.125	57.7	81.0	75.2	69.4	0.398	0.653	0.753
PSNP	0.5	32	55.8	78.1	71.8	66.9	0.347	0.628	0.724
3SPSDP	0.0625	1	58.2	72.4	67.8	65.3	0.309	0.626	0.694
PseDNC + 1SNPF	0.25	0.25	61.5	78.8	74.4	70.1	0.409	0.673	0.759
PseDNC + PSNP	1	64	60.0	80.6	75.7	70.5	0.419	0.672	0.769
PseDNC + 3SPSDP	0.25	2	63.4	78.7	74.9	71.1	0.426	0.686	0.773
PseDNC + 4NF	0.25	1	61.0	79.7	75.0	70.3	0.414	0.673	0.763
PseDNC + CPD	16	16	61.0	78.7	74.1	69.7	0.404	0.669	0.753
PseDNC + 3SPSDP + 4NF	0.25	1	65.1	77.3	74.2	71.2	0.427	0.693	0.777
PseDNC + 3SPSDP + 1SNPF	0.25	0.5	65.2	77.3	74.1	71.2	0.428	0.694	0.776
PseDNC + 3SPSDP + PSNP	0.25	1	64.1	76.8	73.4	70.4	0.412	0.684	0.768
PseDNC + 3SPSDP + CPD	16	16	61.0	78.8	74.2	69.9	0.404	0.670	0.753
PseDNC + 3SPSDP + 4NF + 1SNPF	0.5	2	64.9	78.1	74.7	71.5	0.433	0.695	0.779
PseDNC + 3SPSDP + 4NF + PSNP	0.5	2	63.5	78.5	74.7	71.0	0.424	0.686	0.772
PseDNC + 3SPSDP + 4NF + CPD	16	16	61.0	78.8	74.2	69.9	0.404	0.670	0.755
PseDNC + 3SPSDP + 4NF + 1SNPF + PSNP	0.25	0.5	68.1	75.5	73.5	71.8	0.437	0.707	0.782
PseDNC + 3SPSDP + 4NF + 1SNPF + CPD	16	16	61.1	78.9	74.3	70.0	0.406	0.670	0.756
PseDNC + 3SPSDP + 4NF + 1SNPF + PSNP + CPD	16	16	61.1	78.9	74.3	70.0	0.406	0.670	0.756

### Feature subsets selected by SFS

Considering the fact that different features may be complementary, combination of the six generated features may improve the predictive performance. However, there are also redundancy between these features, and a high dimensional input feature can make the model training very time-consuming and easily over-fitting. In order to solve the problem, we have used the sequential forward feature selection (SFS) strategy to select a compact feature subset from these features to build our final models.

As shown in Table 2, the cross validation accuracy was convergent at the fourth round in the SFS process for training the model of *H. sapiens.* The highest AUROC is 0.899, and the corresponding feature subset includes PSNP, 4NF, 5SNPF and PseDNC.

For *M. musculus*, the cross validation accuracy was convergent at the third round in the SFS process (Table 3). The highest AUROC is 0.822 and the corresponding feature subset includes CPD, 4NF and 1SPSDP.

As for *A. thaliana*, Table 4 shows that the cross validation accuracy is convergent at the fifth round in the SFS process. The highest AUROC is 0.782 and the corresponding feature subset includes PseDNC, 3SPSDP, 4NF, 1SNPF and PSNP.

### Model sensitivity to the selection of negative samples

To evaluate if the selection of the negative samples affects the predictive performances of the models, we built other nine models based on positive samples and other nine negative subsets for both *H. sapiens* and *M. musculus* with the optimal feature subsets. Additional file 1: Tables S1 and S2 show cross-validation performances of the 10 models built on the positive samples and the 10 negative subsets for *H. sapiens* and *M. musculus*, respectively. The means and the standard errors of the ROC AUCs of the ten models are 0.843 and 0.029, and 0.822 and 0.003, for *H. sapiens* and *M. musculus,* respectively. Additional file 1: Tables S3 and S4 show the performances of the ten models on the independent test sets of *H. sapiens* and *M. musculus,* respectively. The means and the standard errors of the ROC AUCs of the ten models are 0.834 and 0.024, and 0.776 and 0.007, for *H. sapiens* and *M. musculus*, respectively. The results indicate the performance of the models is affected a little for *H. sapiens* by the selection of negative samples, however, the performance is barely affected for *M. musculus* by the selection of negative samples. The main possible reason is that the dataset for *H. sapiens* is smaller compared with that of *M. musculus*. The distribution is easily fluctuated for small samples.

### Comparison with other classifiers

Studies above has showed that support vector machine performed well in predicting m5C sites for different species. In order to further investigate and compare the performance of other classifiers, we used other five classifiers, namely KNN [29], Adaboost [30], random forests [31], decision tree [32], logistic regression [33] and XGBoost [34] to build models based on the selected feature subsets for all the three species. The hyper parameters for KNN, Adaboost, random forests and XGboost were also optimized with grid search. The $k$ of KNN is set from 1 to 10 with a step 1. The ntree of 10 to 1000 with a step 20 is set for both Adaboost and random forests. The learning rate, max depth and nrounds of XGboost are set between 2^−4^ and 2^−1^, 2 and 10, and 2^3^ and 2^10^, respectively. Table 5 shows the cross validation results of the six classifiers. For *H. sapiens*, the AUCROC value of SVM is 0.899, which is higher than those of XGBoost, RF, KNN, AdaBoost, decision tree and LR at 0.020, 0.050, 0.049, 0.039, 0.221 and 0.282, respectively. Moreover, the SVM model achieved the highest values in all other metrics. For *M. musculus*, the AUCROC value of SVM is 0.822, which is again higher than those of RF, KNN, AdaBoost, decision tree and LR at 0.008, 0.093, 0.010, 0.207 and 0.011, respectively, but a little bit less than XGBoost (0.823). For *A. thaliana*, SVM again gave the highest AUC value at 0.782, which is higher than those of XGBoost, RF, KNN, AdaBoost, decision tree and LR for 0.012, 0.004, 0.048, 0.026, 0.195 and 0.052, respectively. For other metrics, KNN has the highest Sp value and RF has the highest Pre value, while the Sn, Acc, MCC and F1 score value of SVM offered the highest values for all the remaining 4 metrics. The fact that SVM has outperformed all other six classifiers for both *H. sapiens* and *A. thaliana* and is comparable to XGBoost for M. musculus further confirms that SVM is a stable and robust classifier. As a result, SVM was selected as the final classifier in this study.

**Table 5:** Comparison of different classifiers based on the cross-validation results on the training datasets for the three species

Species	Classifiers	Sn (%)	Sp (%)	Pre (%)	Acc (%)	Mcc	F1score	AUROC
*H. Sapiens*	SVM	**85.5**	**80.0**	**81.0**	**82.8**	**0.656**	**0.832**	**0.899**
	XGBoost	82.5	79.5	80.1	81.0	0.620	0.813	0.879
	RF	77.5	77.0	77.1	77.3	0.550	0.773	0.849
	KNN	84.5	72.5	75.5	78.5	0.574	0.797	0.850
	Adaboost	79.5	73.5	75.0	76.5	0.530	0.772	0.860
	DT	68.0	65.0	66.1	66.5	0.330	0.670	0.678
	LR	62.0	61.5	61.7	61.8	0.235	0.618	0.617
*M. musculus*	SVM	75.7	72.8	73.6	74.3	0.486	0.746	0.822
	XGBoost	**76.1**	**73.6**	**74.3**	**74.9**	**0.498**	**0.752**	**0.823**
	RF	75.9	71.6	72.8	73.7	0.476	0.743	0.814
	KNN	67.3	67.5	67.5	67.4	0.349	0.674	0.729
	Adaboost	74.2	72.6	73.0	73.4	0.468	0.736	0.812
	DT	62.6	62.3	62.4	62.5	0.250	0.630	0.615
	LR	73.3	73.2	73.2	73.2	0.465	0.733	0.811
*A. thaliana*	SVM	**68.1**	75.5	73.5	**71.8**	**0.437**	**0.707**	**0.782**
	XGBoost	65.1	76.3	73.3	70.7	0.417	0.690	0.770
	RF	66.1	76.8	**74.1**	71.5	0.432	0.699	0.778
	KNN	58.0	**78.6**	73.1	68.3	0.375	0.647	0.734
	Adaboost	65.2	74.2	71.6	69.7	0.395	0.683	0.756
	DT	59.5	60.0	59.8	59.8	0.200	0.600	0.587
	LR	64.4	69.8	68.1	67.1	0.342	0.662	0.730

Bold numbers indicate the highest values in each column for different species

### Comparison with other existing methods

**Fig. 2:**
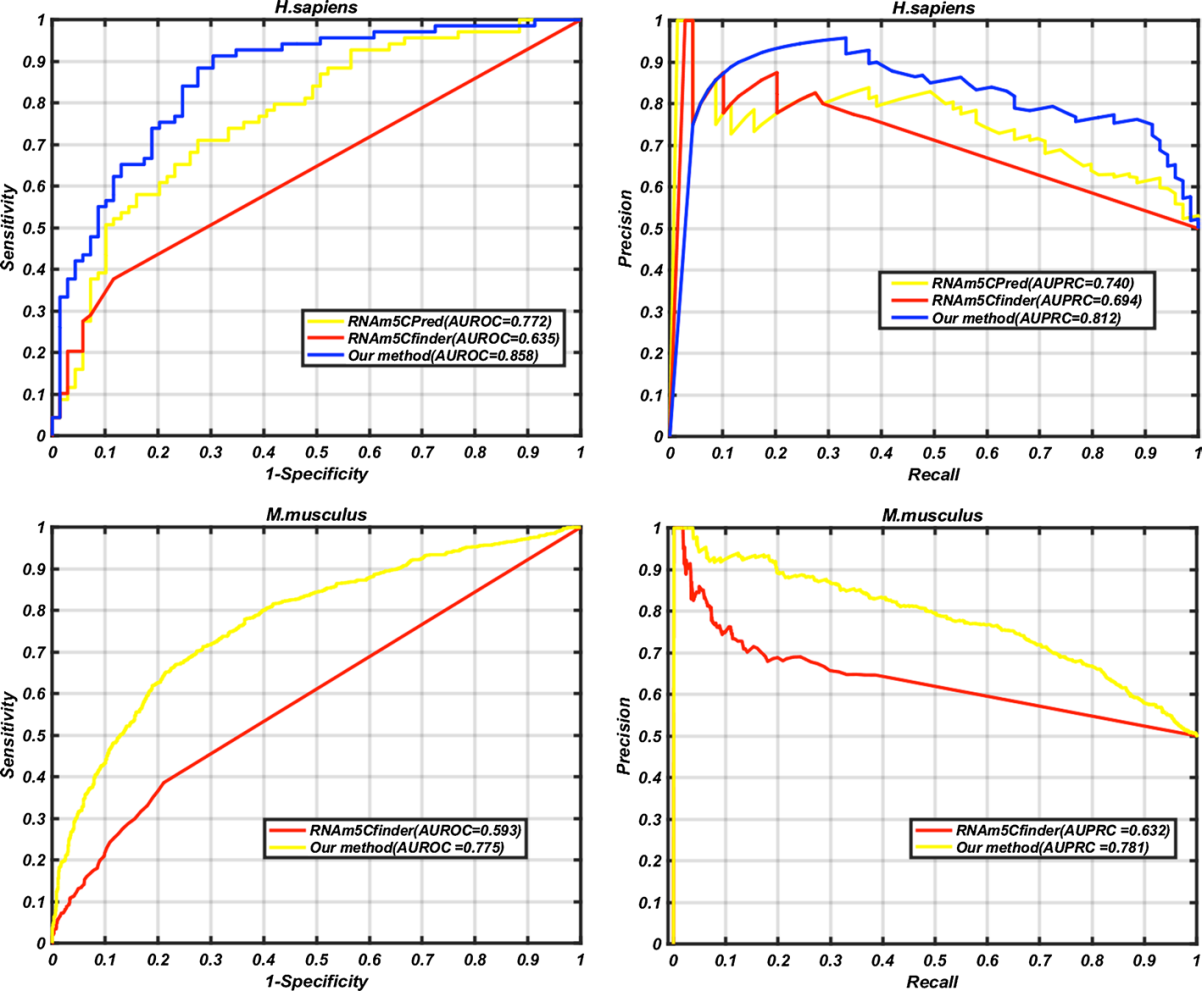
ROC curves and PRC curves for our model and other models on the independent test sets. Upper panel: *H. sapiens*; Lower panel: *M. musculus*

In this study, we have also compared our methods with some other existing m5C site prediction methods [19–26]. Because different benchmark datasets have been used for building different methods, independent test sets were used to ensure the objectiveness of the comparison. These independent test sets were only used for comparison and not for building our models. At present, four methods are available to identify m5C sites of *H. sapiens*, namely RNAm5Cfinder [25], iRNA-m5C [26], iRNAm5C-PseDNC [20] and RNAm5CPred [23]. Two methods are available for predicting m5C sites of *M. musculus,* namely RNAm5Cfinder [25] and iRNA-m5C [26]. Two methods are available for detecting m5C sites of *A. thaliana*, namely PEA-m5C [24] and iRNA-m5C. Table 6 shows the predictive results of these methods on the independent test sets for the three species, and Fig. 2 shows the relevant ROC curves and PRC curves. For *H. sapiens*, iRNAm5C-PseDNC has the highest Sp value (0.971), while our method gives significantly higher values for Sn, Pre, Acc, MCC, F1 score and AUROC when compared with other methods. For *M. musculus*, other than Sp and Pre, again our method has the highest values for all the remaining metrics (Sn, Pre, Acc, MCC, F1 score and AUROC). For *A. thaliana,* our method gives the highest value for all the metrics. All these results have indicated that our methods performed better than other existing methods in predicting m5C sites.

**Table 6:** Comparison with existing methods on the independent test sets

Species	Model	Sn (%)	Sp (%)	Pre (%)	Acc (%)	Mcc	F1-score	AUROC^a^
*H. sapiens*	RNAm5Cfinder	37.7	88.4	76.5	63.1	0.303	0.505	0.635
	iRNA-m5C	42.1	46.4	43.9	44.2	-0.116	0.429	–
	iRNAm5C-PseDNC	4.35	**97.1**	60.1	50.7	0.039	0.081	–
	RNAm5CPred	71.0	66.7	68.1	68.9	0.377	0.695	0.772
	our method	**75.4**	79.7	**78.8**	**77.5**	**0.551**	**0.77**	**0.858**
*M. musculus*	RNAm5Cfinder	38.6	78.9	64.5	58.8	0.191	0.483	0.593
	iRNA-m5C	0.61	**99.8**	**75.1**	50.2	0.032	0.012	–
	our method	**67.9**	74.9	73.0	**71.4**	**0.429**	**0.704**	**0.775**
*A. thaliana*	iRNA-m5C	72.4	75.6	73.5	74.1	0.481	0.729	–
	PEA-m5C	43.2	45.4	43.8	44.3	-0.114	0.454	–
	our method	**75.5**	**76.1**	**76.0**	**75.8**	**0.516**	**0.757**	**0.836**

Bold numbers indicate the highest values in each column for different species

^a^There are no predicted scores of iRNA-m5C, iRNAm5C-PseDNC and PEA-m5C, so the AUROCs for these methods were not available

### Cross-species verification

**Fig. 3:**
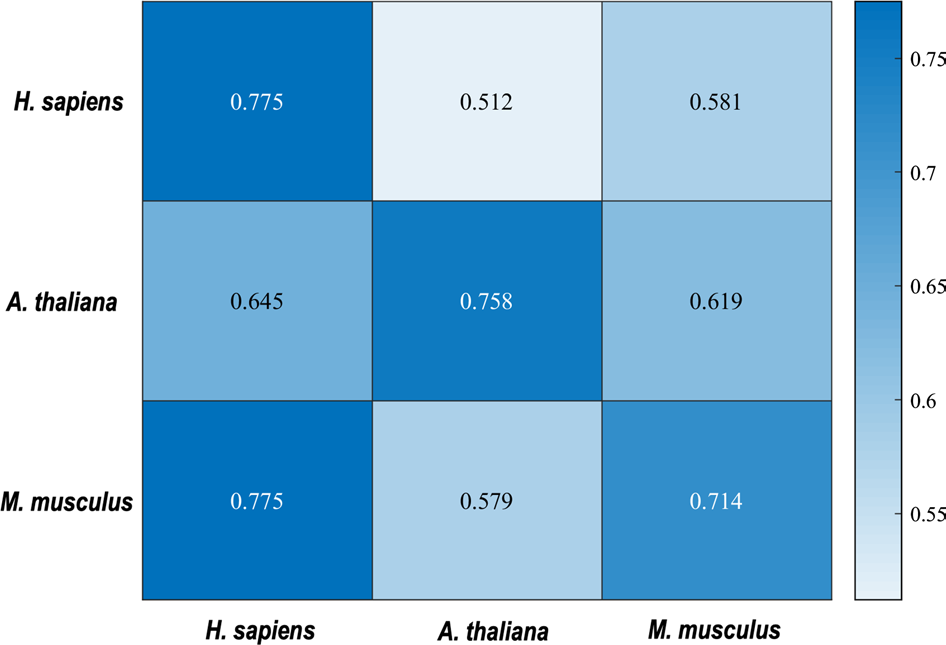
The heat map showing the cross species prediction accuracies. Once a species-specific model was established on its own training dataset in rows, it was validated on the data from the same species as well as the independent data from the other two species in columns

**Fig. 4:**
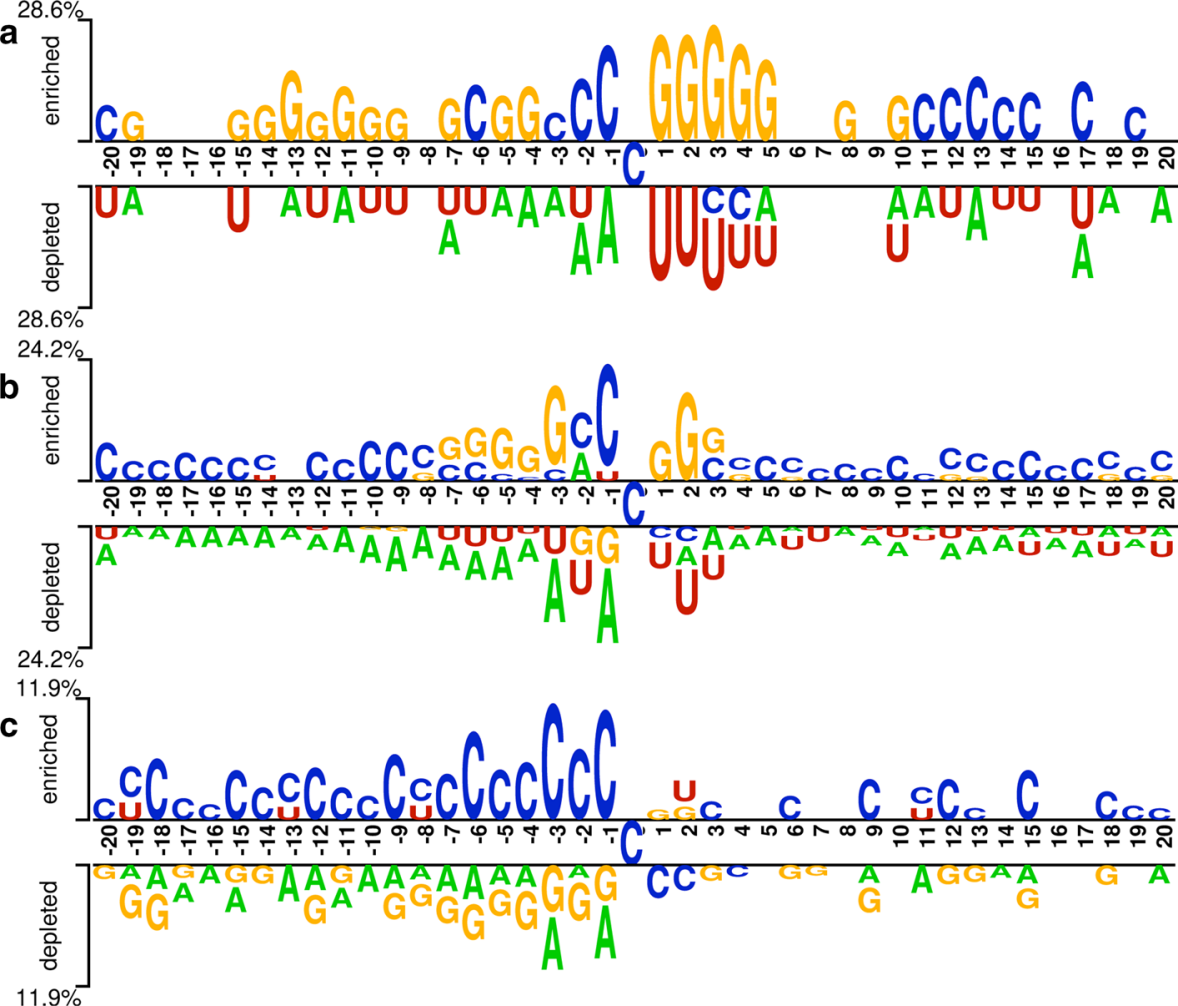
The nucleotide distribution around m5C and non-m5C sites. The top panel of the x-axis is for m5C site containing sequence, while the down panel of the x-axis is for non-m5C site containing sequences. **a** *H. sapiens*; **b** *M. musculus;* **c** *A. thaliana*

In this study, models were built for *H. sapiens*, *M. musculus* and *A. thaliana* individually. It will be of great interest to evaluate the species-specificity and transferability of these models using the cross-species verification. To achieve that, the three models built on the three species-specific m5C training data sets were further tested on the three independent test datasets. Figure 3 shows the test results. Firstly, all the three models performed well on its own independent test sets (see the diagonal of Fig. 3). Secondly, the models of *H. sapiens* and *M. musculus* both performed poorly on the independent test set of *A. thaliana*, and vice versa. One possible reason to explain this result is because *H. sapiens* and *M. musculus* are both mammals while *A. thaliana* is plant. This reason is supported by Fig. 4, which shows that the nucleotide distribution in the sequence of A*. thaliana* is different from that of *H. sapiens* and *M. musculus*. Thirdly, the M. musculus-specific model performed well on the *H. sapiens*-specific independent test set, however, the *H. sapiens*-specific model performed poorly on the *M. musculus*-specific independent test set. It might be because the *H. sapiens*-specific datasets have smaller sizes than the *M. musculus*-specific datasets, and the smaller datasets sizes limit the variety of sequences which confines the transferability of the *H. sapiens*-specific model.

### Web implementation

For the convenience of researchers, a user-friendly and publicly accessible web server was built to implement our method, which is available at https://zhulab.ahu.edu.cn/m5CPred-SVM. Users can predict the m5C sites on this server without complicated calculation. The detailed procedure to use the web server is as below:

To start with, users need to choose one from the three species, *H. sapiens*, *M. musculus* and *A. thaliana.* After that, users can type the query RNA sequences into the input box or upload a FASTA format file (Note that the input sequence should be in FASTA format and the length of each query sequence should be longer than 41 bp). Then, by clicking the 'submit' button, the system will do the calculation and give the final result. In the backend, the server would find the cytosine in the query sequence. All cytosine-centric RNA fragment would be extracted with flank size equals to 20 and the missing nucleotides would be filled by ‘N’. There might be lots of cytosines in a sequence, and our predictive model will reconstruct the sequence separately for each of them. The server home page also contains our contact information for users to contact us in case they have problems with the server or have suggestions.

## Discussion

Our study shows that the position specific related features can be effective features for discriminating m5C sites from non-m5C sites. Theoretically speaking, the difference of nucleotide distribution between RNA sequences containing m5C sites and those without m5C sites determines how well we can discriminate them. In other words, the nucleotide distribution around the m5C site may have a certain preference. In order to investigate the nucleotide distribution preference for each sequence position, we adopted Two Sample Logo tool [35] to conduct visualization of the nucleotide site preference around m5C and non-m5C sites in the three species. Figure 4 clearly shows that significant difference does exist in nucleotide distribution around the m5C sites and the non-m5C sites for these three species, and the difference was found to descend in the sequence of *H. sapiens*, *M. musculus* and *A. thaliana* according to the depleted ratio (see Y axis of Fig. 4). It is shown that the depleted ratio of *H. sapiens* is from −28.6 to 28.6% and the depleted ratio of *M. musculus* is from −24.2 to 24.2%, which means the differences of nucleotide position preferences between positive and negative samples of the two species are significantly different. However, the corresponding depleted ratio of *A. thaliana* is from −11.9 to 11.9%. This is in line with our results that the six types of features for *H. sapiens* performed better than those for *M. musculus*, and the features for *A. thaliana* performed worst. The sequence differences observed here may account for the performance difference of the six types features observed before for the three species.

In addition, this figure can also explain why PSNP, KSPSDP performed well among the six types of features for both *H. sapiens* and *M. musculus*, while PseDNC and 4NF achieved best accuracy for *A. thaliana*. Among these six types of features, PSNP and KSPSDP are the two features that consider position preference information. As mentioned before, both *H. sapiens* and *M. musculus* have high position preferences of nucleotide in RNA sequences, thus it is not surprising that PSNP and KSPSDP performed best for these two species. On the contrary, the position preferences of RNA sequences of *A. thaliana* are not as significant as those of *H. sapiens* and *M. musculus*, so the two features, PSNP and KSPSDP, did not performed as well as they did for *H. sapiens* and *M. musculus*.

KNF, KSNPF and pseDNC are three features related to nucleotide composition of RNA segments. KNF can describe the local sequence-order information of nucleotide sequences. The idea of KSNPF is to calculate the frequency of sixteen pairs of nucleotides spaced by K-length polynucleotides. With increasing of K, KSNPF feature takes the position correlation information into account within the nucleotide sequence. PseDNC feature contains both local and global sequence-order information. The performances of these three features are determined by the composition difference between positive and negative samples.

The CPD feature contains nucleotide information at each position of the RNA segments and it also contains the nucleotide composition information along the RNA sequences, so that it performs well for all these three species.

According to our results, models based on these selected feature subsets selected by SFS had made improvements of about two percents in performance when compared with models based on single feature. As all of these selected feature subsets are combinations of the position specific features and the composition features, the improvements observed here can further confirm the complementarity between these two groups of features. It should be noted that we tried a large number of other types of features generated by iLearn [36] or Pse-in-One [37] toolkits when we designed the input features (data not shown). The sequence-based features generated by these two toolkits have been used widely for predicting both RNA post-transcriptional modification sites [38–40] and post-translational modification sites [41, 42]. Our experimental results demonstrated that our proposed feature combination in this study yielded satisfactory performance, which cannot be significantly improved when they were combined with other features.

We summarized the possible reasons for our method to outperform other existing methods. For the benchmark datasets, we used larger training sets for *H. sapiens* and *M. musculus* than iRNA-m5C which is the latest model for multiple species. Large datasets are helpful for improvement of the generalization of models. In addition, we added two types of position specific propensity features, PSNP and KSPSDP. Our results (Tables 2, 3 and 4) demonstrate PSNP and KSPSDP have played key roles in improving method performance.

## Conclusion

In this study, a new computational method, m5CPred-SVM, was developed for predicting m5C sites in RNA sequences. Non-redundant large benchmark datasets were collected for three species, namely *H. sapiens*, *M. musculus* and *A. thaliana*. A total of six types of features, including features related to composition, features related to position specific and features related to physicochemical properties were used in building our models. Results have showed that the features related to position specific are effective in differentiating m5C sites from non-m5C sites for *H. sapiens* and *M. musculus*. Nucleotide distribution analysis reveals that nucleotide position preferences are significant for both *H. sapiens* and *M. musculus*, which account for the effectiveness of the features related to position specific propensity. For the same reason, the features related to position specific propensity are not that effective for *A. thaliana* because the nucleotide position preferences are less significant compared with that for the other two species. Optimal feature subsets were selected from these six types of features using the sequential forward feature selection strategy. All the three subsets consisted of feature related position specific propensity and feature related to nucleotide composition which indicate the complementarity between the features. The performance of our method m5CPred-SVM was objectively compared with other existing methods by using independent test sets. The results showed that our method can offer significantly better performances than all the other existing methods. Finally, a web server was built at https://zhulab.ahu.edu.cn/m5CPred-SVM to facilitate the access to our method by academic users to predict the m5C sites in RNA sequences.

## Methods

### Benchmark datasets

High quality benchmark datasets are extremely important for training and evaluating machine learning models. In this study, m5C data of three species have been collected from recently published literature. For *A. thaliana*, same datasets constructed by Lv et al. [26] were used for fair comparison. The positive RNA segments which contain m5C site in the center were collected from NCBI Gene Expression Omnibus (GEO) database with the accession number GSE94065 [43]. This dataset contains 6289 positive samples and 6289 negative samples.

The positive samples in the datasets of *M. musculus* and *H. sapiens* were obtained from the works of Yang et al. [44] and Vahid Khoddami et al. [18], respectively. For *H. sapiens*, we collected the data from the work of Vahid Khoddami et al. [18]. The file “GSE90963_Table_S1-m5C_candidate_sites.xlsx” was downloaded from GEO (https://www.ncbi.nlm.nih.gov/geo/query/acc.cgi?acc=GSE90963), which recorded both m5C sites information by their RBS-seq work and the m5C sites information in other public datasets. Firstly, we collected the sites with high-threshold in their RBS-seq work. Secondly, we collected the sites both reported in their RBS-seq work and the public datasets. Totally, 408 m5C sites were collected for *H. sapiens*. For *M. musculus*, we collected the data from Additional file 1: Table S3 of Yang et al.’s work [44]. The m5C sites detected in six different tissues are all considered as positive examples, thus we obtained 13042 RNA segments centered with m5C. In order to avoid bias of the datasets, similar sequences in the datasets were removed using the CD-HIT program [45] with the sequence identity threshold set at 70%, through which we have obtained 5563 and 269 positive samples for *M. musculus* and *H. sapiens*, respectively. In machine learning, the model performance may be degraded and the prediction results may be out of balance due to the inconsistency of the amount of data between the positive sample and the negative sample [46, 47]. Therefore, we have randomly selected the same number of negative samples as that of positive samples for the establishment of the benchmark dataset. It is worth noting that the redundancy of the negative examples was also removed using CD-HIT with the sequence identity threshold set at 70%. To verify if the model is sensitive to the selection of negative samples, we conducted the same procedure to generate other nine negative subsets for both *H. sapiens* and *M. musculus*. We have not done the same thing for *A. thaliana* because we did not know the details about the generation of negative samples of *A. thaliana* which were obtained from Lv et al.’s work.

The benchmark dataset is usually divided into two parts. One is the training dataset and the other is the independent test set. The training dataset is used for model construction, cross-validation and determination of hyper-parameters of the learning algorithms. The independent test dataset is used to test the performance and generalization ability of the model. In this study, 69 positive samples and 69 negative samples were randomly selected as the independent test dataset and the remaining 200 positive samples and 200 negative samples were used as the training dataset for *H. sapiens*. For *M. musculus* and *A. thaliana*, 1000 positive samples and 1000 negative samples were randomly selected as the independent test datasets, and the remaining samples (4563 positive samples and 4563 negative samples for *M. musculus*, 5298 positive samples and 5298 negative samples for *A. thaliana*) were used as the training datasets.

The fragment of each RNA in the datasets is represented as:

1 $$R_{\lambda } \left( C \right) = N_{ - \lambda } N_{{ - \left( {\lambda - 1} \right)}} \ldots N_{ - 1} CN_{1} \ldots N_{{ + \left( {\lambda - 1} \right)}} N_{\lambda }$$

where $N_{ - \lambda }$ represents the upstream nucleotide of central cytosine and $N_{\lambda }$. rresents the downstream nucleotide of central cytosine. In most previous works [19–22, 26], the length of the input RNA segments was set to 41 and the m5C site is located in the central position 21. In this study, we have also extracted features from the 41 bp long RNA segments.

The details of the training dataset and the testing dataset are shown in Table 7.

**Table 7:** The information of the datasets

Dataset^a^	Length (bp)	Positive subset	Negative subset	Total
H_train	41	200	200	400
H_test	41	69	69	138
M_train	41	4563	4563	9126
M_test	41	1000	1000	2000
A_train	41	5289	5289	10,578
A_test	41	1000	1000	2000

^a^ ‘H’ represents *H. sapiens*, ‘M’ represents *M. musculus* and ‘A’ represents *A. thaliana*

### Feature extraction

#### K-nucleotide frequency (KNF)

As a classic sequence coding feature, K-nucleotide frequency (KNF, also called NC (Nucleotide composition)) has been widely used to build bioinformatics models [48–50]. Suppose we have an RNA segment R of length L:

2 $$R = n_{1} n_{2} n_{3} \ldots n_{i} \ldots n_{L - 1} n_{L}$$

$n_{i}$ indicates the *i*th nucleotide of R, and it can be any one of the four nucleotide bases in RNA, i.e.$n_{i}$ $\in${A, C, G, U}. For a given K value, KNF represents the frequency of occurrence of each K-mer nucleotide component in the nucleotide sequence. It can be calculated by the formula (3).

3 $$f\left( {n_{1} n_{2} \ldots n_{k} } \right) = \frac{{N\left( {n_{1} n_{2} \ldots n_{k} } \right)}}{L - K + 1}$$

where $n_{1} n_{2} \ldots n_{K}$ indicates a K-mer nucleotide component. It is not difficult to find that the K-mer nucleotide composition of an RNA sequence is a $4^{K}$-dimensional vector consisting of frequency of each K-mer type. As the value of K increases, the dimension of the feature vector increases exponentially. For example, when K = 1, four types of single nucleotide frequencies can be obtained. We chose the K value of 4 to calculate the frequency at which 4 nucleotides appears (4NF) according to a previous work [23]. The RNA fragment can be encoded as:

4 $$R\left( {4NF} \right) = \left[ {f_{AAAA} f_{AAAC} \ldots f_{GCUU} \ldots f_{UUUG} f_{UUUU} } \right]$$

#### K-spaced nucleotide pair frequency (KSNPF)

K-spaced nucleotide pair frequency is another method for encoding RNA sequences [51]. This method mainly calculates the frequency of 16 pairs of nucleotides separated by k-length polynucleotides. We use $n_{1}$ × {K}$n_{2}$ to represent K-spaced nucleotide pairs. Since $n_{1}$ and ${ }n_{2} { }$ have four possible values, so there are sixteen ($4^{2}$ = 16) possible combinations. For example: AxxC is a two spacer nucleotide pair. The calculation formula of KSNPF is

5 $$f\left( {n_{1} {\text{x}}\left\{ {\text{K}} \right\}n_{2} } \right) = \frac{{N\left( {n_{1} {\text{x}}\left\{ {\text{K}} \right\}n_{2} } \right)}}{L - K + 1}$$

In this work, we tried different K values in order to determine the best KSNPF features for different species. The selection of K for different species can be found in Additional file 1: Table S5.

#### Position-specific nucleotide propensity (PSNP)

In several previous works [27, 28, 51], position-specific nucleotide propensity has been used to predict the post-transcriptional modification of RNA. This feature is obtained by calculating the difference in nucleotide frequencies at specific positions between positive and negative RNA fragments. It was first introduced in Li et al.’s work [28]. According to Eq. (1), the RNA fragment can be re-expressed as:

6 $$R_{\lambda } = N_{1} N_{2} \ldots N_{i} \ldots N_{2\lambda + 1}$$

First, we calculated the frequency of the four nucleotides at the *i*-th position in the positive sample and the negative sample, respectively. After that, the 4-dimensional positive vectors and the 4-dimensional negative vectors were combined individually to obtain two $4 \times \left( {2\lambda + 1} \right)$ position-specific occurrence frequency matrices for positive and negative samples, respectively. The two matrices were named as $M^{ + }$ and $M^{ - }$,$M^{ + }$ is for positive samples and $M^{ - }$ is for negative samples. Through $M^{ + }$ and $M^{ - }$,we defined the position-specific nucleotide propensity matrix, denoted as $X_{PSNP}$, as below:

7 $$X_{PSNP} = M_{ + } - M_{ - }$$

#### K-spaced position-specific dinucleotide propensity (KSPSDP)

Position-specific dinucleotide propensity is defined using the similar procedure to define PSNP. To calculate this feature, we rewrite Eq. (6) as a dinucleotide:

8 $$R_{\lambda } = D_{1} D_{2} \ldots D_{i} \ldots D_{2\lambda }$$

where *D*_*i*_ represents the dinucleotide at the *i*-th position of RNA and has 16 types of values. By using the similar way for calculating the PSNP feature, we can get the ($16 \times 2\lambda$) position-specific dinucleotide propensity (PSDP) matrix.

To calculate K-spaced position-specific dinucleotide propensity, $n_{1}$ × {K}$n_{2}$ was used to represent K-spaced nucleotide pairs. PSDP is a specific case for KSPSDP when K equals 0. In this work, we tried different K values to determine the best KSPSDP features for different species. The selection of K values for different species can be found in Additional file 1: Table S6.

#### Pseudo dinucleotide composition (PseDNC)

The pseudo K-tuple nucleotide composition (PseKNC) has been used to represent an RNA sequence with a discrete model or vector which can keep considerable sequence order information, especially the global or long-range sequence order information [20, 26, 52, 53]. In this study, we used PseDNC (K = 2 for PseKNC) to encode the RNA segments. Three physicochemical properties, free energy, hydrophilicity and stacking energy were used to generate features of PseDNC. The values of these three physicochemical properties of 16 dinucleotides are shown in Table 8.

**Table 8:** Three types of physicochemical properties of dinucleotides in RNA

Dinucleotide	Free energy	Hydrophilicity	Stacking energy
GG	−3.260	0.170	−11.100
GA	−2.350	0.100	−14.200
GC	−3.420	0.260	−16.900
GU	−2.240	0.270	−13.800
AG	−2.080	0.080	−14.000
AA	−0.930	0.040	−13.700
AC	−2.240	0.140	−13.800
AU	−1.100	0.140	−15.400
CG	−2.360	0.350	−15.600
CA	−2.110	0.210	−14.400
CC	−3.260	0.490	−11.100
CU	−2.080	0.520	−14.000
UG	−2;.110	0.340	−14.400
UA	−1.330	0.210	−16.000
UC	−2.350	0.480	−14.200
UU	−0.930	0.440	−13.200

#### Chemical property with density (CPD)

The four types of nucleotides in RNA (A (adenine), U (uracil), G(guanine) and C(cytosine)) can be divided into three categories according to their chemical structures and internal binding characteristics [54]. Considering the ring structure of the nucleotide, C and U are pyrimidines with one ring, while A and G are purines with two rings. As for the secondary structure, the hydrogen bonds of A and U are weak, while the hydrogen bonds of G and C are strong. In terms of chemical functionality, U and G are classified as keto groups, while A and C are in amino groups. These three aspects of chemical properties can be represented as a three-dimensional vector (*x*, *y*, *z*), where *x,* *y,* *z* represent the ring structure, the hydrogen bond, and the chemical functionality of the nucleotides respectively. In this way, each nucleotide $n_{i} = \left( {x_{i} ,y_{i} ,z_{i} } \right)$ in an RNA sequence can be encoded as:

9 $$x_{i} = \left\{ {\begin{array}{*{20}c} {1 if n_{i} \varepsilon \left\{ {A,} \right.\left. G \right\}} \\ {0 if n_{i} \varepsilon \left\{ {U,} \right.\left. C \right\}} \\ \end{array} } \right., y_{i} = \left\{ {\begin{array}{*{20}c} {1 if n_{i} \varepsilon \left\{ {A,} \right.\left. C \right\}} \\ {0 if n_{i} \varepsilon \left\{ {U,} \right.\left. G \right\}} \\ \end{array} } \right., z_{i} = \left\{ {\begin{array}{*{20}c} {1 if n_{i} \varepsilon \left\{ {A,} \right.\left. U \right\}} \\ {0 if n_{i} \varepsilon \left\{ {C,} \right.\left. G \right\}} \\ \end{array} } \right.$$

Thus, the four types of nucleotide, A, U, G and C, can be encoded as (1,1,1), (0,0,1), (0,1,0), (1,0,0), respectively.

In order to better represent the distribution of each nucleotide in the RNA sequence, the density of a nucleotide, which describes the frequency of the nucleotide occurring before current position, is denoted as:

10 $$d_{i} = \frac{1}{{\left| {N_{i} } \right|}}\mathop \sum \limits_{j = 1}^{i} f\left( {n_{j} } \right), f\left( {n_{j} } \right) = \left\{ {\begin{array}{*{20}c} {1 \;if\; n_{j} = p} \\ {0 \;if\; n_{i} \ne p} \\ \end{array} } \right.$$

where *d*_*i*_ is the density of nucleotide, *i* is the current position of RNA sequence, $\left| {N_{i} } \right|$ is the length of the *i*th prefix string $\left\{ {n_{1} ,n_{2} , \ldots ,n_{i} } \right\}$ in the sequence, and $p$ is the symbol of {A, U, G, C}.

By integrating the nucleotide chemical property and the distribution of each nucleotide in the RNA sequence, a ($4 \times \xi$)-dimensional CPD feature vector can be generated, where $\xi$ is the length of the RNA segment.

### Support vector machine

Support vector machine (SVM) is a popular statistical learning method and has been extensively used to build bioinformatics models [23, 50, 55–58] because of its high efficiency and robust output. In this study, we used the MATLAB function FITCSVM to build our models. SVM uses kernel functions to project low-dimensional data into high-dimensional space. A few different kernel functions can be used in training. In this work, the radial basis kernel function was selected with two hyper parameters (box constraint and kernel scale) to be used with FITCSVM function. The two parameters were optimized by a grid search with box constraint from $2^{ - 5}$ to $2^{15}$ and kernel scale from $2^{ - 10}$ to $2^{6}$.

### Evaluation criteria

Ten-fold cross-validation was used to evaluate the generalization performance based on the training dataset. For the ten-fold cross-validation, the training dataset was divided into ten roughly equal-sized subsets with a stratified sampling, and then one subset was used as a validation set whereas the remaining nine subsets were combined for training. This process was repeated ten times with ten models built and validated. Finally, the average performance was obtained. In this study, the ten-fold cross-validation was used for feature selection, parameter optimization and classifier comparison.

Different metrics were used to assess the model performance, namely accuracy (Acc), sensitivity (Sen), specificity (Spe), precision (Pre), Matthews correlation coefficient (Mcc) and F1-score. The specific formulas are as below:

11 $$\left\{ {\begin{array}{*{20}c} {{\text{Sen}} = \frac{{{\text{TP}}}}{{{\text{TP}} + {\text{FN}}}}} \\ {{\text{Spe}} = \frac{{{\text{TN}}}}{{{\text{TN}} + {\text{FP}}}}} \\ {{\text{Pre}} = \frac{{{\text{TP}}}}{{{\text{TP}} + {\text{FP}}}}} \\ {{\text{Acc}} = \frac{{{\text{TP}} + {\text{TN}}}}{{{\text{TP}} + {\text{FN}} + {\text{FP}} + {\text{FN}}}}} \\ {{\text{Mcc}} = \frac{{{\text{TP }} \times {\text{*TN}} - {\text{FP* FN}}}}{{\sqrt {\left( {{\text{TP}} + {\text{FP}}} \right)\left( {{\text{TP}} + {\text{FN}}} \right)\left( {{\text{TN}} + {\text{FP}}} \right)\left( {{\text{TN}} + {\text{FN}}} \right)} }}} \\ {{\text{F}}1 = \frac{2*TP}{{\left( {2*TP + FP + FN} \right)}}} \\ \end{array} } \right.$$

where, TP, TN, FP and FN represent the number of true-positive (m5C sites that were predicted as m5C sites), true-negative (non-m5C sites that were predicted as non-m5C sites), false-positive (non-m5C sites that were predicted as m5C sites) and false-negative (m5C sites that were predicted as non-m5C sites) samples, respectively.

In addition, we draw the receiver operating characteristic curve (ROC curve) [59] and precision recall curve (PRC curve) [60], to evaluate the performances of different models. ROC curve demonstrates the relationship between sensitivity and 1-specificity at different thresholds, and PRC curve reflects the trend of precision changing with recall. These two curves can be used to evaluate the predictive capability of the proposed method across entire range of decision values. The areas under these two curves (AUROC and AUPRC) were also calculated to quantify the model performance. AUROC and AUPRC have value ranging from 0 to 1. The closer the value approximate 1, the better the model performance is.

### Feature selection

There are three major methods for feature selection: Filter, Wrapper and Embedded. We have chosen the sequence forward selection algorithm (SFS) under Wrapper as the feature selection algorithm in this study. Six types of features are generated and constitute the high-dimensional feature vector of each sample. The following specific operations of SFS were used to achieve a compact and efficient feature subset: in the first round, the ten-fold cross-validation results were obtained for models built on each of the six types of features. The best performing feature type was selected according to the AUROC value and then proceeded to the next round of calculation. In the second round, the remaining five types of features were added to the best performing feature type selected in the first round. Similarly, the best performing feature combination was again selected according to the AUROC value and proceeded to the next round of calculation. This process continued until AUROC converged. The subset with the highest AUROC value was considered as the optimal feature subset.

**Fig. 5:**
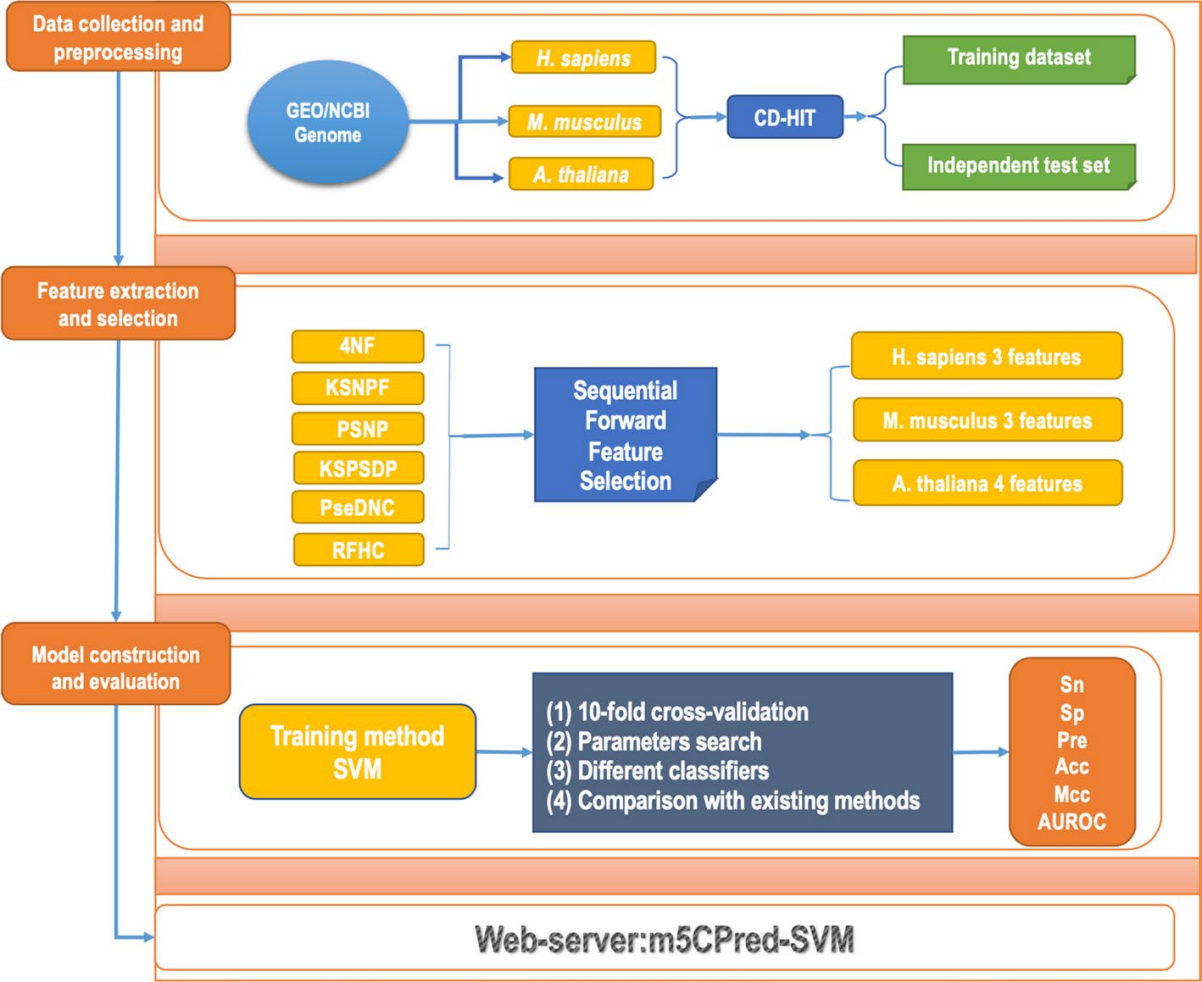
The flowchart of m5CPred-SVM

The entire procedure of m5CPred-SVM is illustrated in Fig. 5.

## Supplementary information

**Additional file 1**. This file provides the performances of models built on positive samples and other nine negative subsets for* H. sapiens* and* M. musculus*, and more detailed data for selecting the Ks for KSNPF and KSPSDP.** Table S1**: The cross validation performances using different negative subsets of * H. sapiens*.** Table S2**: The cross validation performances using different negative subsets of* M. musculus*.** Table S3**: The performances on the independent test sets for models built on different negative subsets of * H. sapiens*.** Table S4**: The performances on the independent test sets for models built on different negative subsets of * M. musculus*.** Table S5**: The cross validation results of KSNPF for the three species with different Ks.** Table S6**: The cross validation results of KSPSDP for the three species with different Ks.

## Data Availability

The webserver is at https://zhulab.ahu.edu.cn/m5CPred-SVM/. The data sets used in this study are also available on the website. All other data generated or analyzed during this study are included in this published article or the Additional files.

## Abbreviations

SVM: Support vector machine
Sn: Sensitivity
Sp: Specificity
Acc: Accuracy
Pre: Precision
Mcc: Matthew correlation coefficient
AUC: Area under the curve
ROC: Receiver operating characteristic
SFS: Sequential forward feature selection
