# Supplementary material for: m5CPred-SVM: a novel method for predicting m5C sites of RNA
Source: BMC Bioinformatics. 2020 Oct 30;21:489. doi: 10.1186/s12859-020-03828-4 (PMC7602301; doi:10.1186/s12859-020-03828-4)
Supplement: Supplementary file 1 — Additional file 1. This file provides the performances of models built on positive samples and other nine negative subsets for H. sapiens and M. musculus, and more detailed data for selecting the Ks for KSNPF and KSPSDP. Table S1: The cross validation performances using different negative subsets of H. sapiens. Table S2: The cross validation performances using different negative subsets of M. musculus. Table S3: The performances on the independent test sets for models built on different negative subsets of H. sapiens. Table S4: The performances on the independent test sets for models built on different negative subsets of M. musculus. Table S5: The cross validation results of KSNPF for the three species with different Ks. Table S6: The cross validation results of KSPSDP for the three species with different Ks. [file 12859_2020_3828_MOESM1_ESM.docx]

Supplementary Materials for

**m5CPred-SVM: A Computational RNA m5C Site Predictor based on Support Vector Machine**

Xiao Chen^1^, Yi Xiong^2^, Yinbo Liu^1^, Yuqing Chen^1^, Shoudong Bi^1^ and Xiaolei Zhu^1^

**^1^** School of Sciences, Anhui Agricultural University, Hefei, Anhui 230036, China

^2^ School of Life Sciences and Biotechnology, Shanghai Jiao Tong University, Shanghai 200240, China

Contact: [xlzhu_mdl@hotmail.com](mailto:xlzhu_mdl@hotmail.com); [bishoudong@163.com](mailto:bishoudong@163.com)

**Table S1.** The cross validation performances using different negative subsets of *H. sapiens.*

| Data_training | Sn% | Sp% | Pre% | Acc% | Mcc | F1-score | AUC |
| --- | --- | --- | --- | --- | --- | --- | --- |
| Pos + Neg1 | 86.0 | 80.0 | 81.0 | 82.8 | 0.656 | 0.832 | 0.899 |
| Pos + Neg2 | 78.0 | 76.0 | 76.4 | 76.8 | 0.535 | 0.769 | 0.853 |
| Pos + Neg3 | 75.0 | 72.0 | 72.8 | 73.5 | 0.470 | 0.739 | 0.816 |
| Pos + Neg4 | 74.0 | 72.0 | 72.6 | 73.0 | 0.460 | 0.733 | 0.824 |
| Pos + Neg5 | 77.0 | 76.0 | 75.7 | 76.0 | 0.520 | 0.761 | 0.845 |
| Pos + Neg6 | 73.0 | 72.0 | 72.1 | 72.3 | 0.445 | 0.723 | 0.805 |
| Pos + Neg7 | 76.0 | 78.0 | 77.2 | 76.8 | 0.535 | 0.766 | 0.842 |
| Pos + Neg8 | 80.0 | 82.0 | 81.5 | 80.8 | 0.615 | 0.805 | 0.881 |
| Pos + Neg9 | 77.0 | 76.0 | 76.1 | 76.3 | 0.525 | 0.763 | 0.825 |
| Pos + Neg10 | 77.0 | 76.0 | 76.2 | 76.5 | 0.530 | 0.766 | 0.839 |
| mean | 77.0 | 75.9 | 76.2 | 76.5 | 0.529 | 0.766 | 0.843 |
| Std error | 3.55 | 3.37 | 3.23 | 3.29 | 0.066 | 0.033 | 0.029 |

**Table S2.** The cross validation performances using different negative subsets of *M. musculus.*

| Data_training | Sn% | Sp% | Pre% | Acc% | Mcc | F1-score | AUC |
| --- | --- | --- | --- | --- | --- | --- | --- |
| Pos + Neg1 | 76.0 | 73.0 | 73.6 | 74.3 | 0.486 | 0.746 | 0.822 |
| Pos + Neg2 | 75.0 | 74.0 | 74.1 | 74.5 | 0.491 | 0.748 | 0.827 |
| Pos + Neg3 | 76.0 | 74.0 | 74.6 | 75.1 | 0.501 | 0.753 | 0.826 |
| Pos + Neg4 | 75.0 | 74.0 | 74.2 | 74.4 | 0.489 | 0.746 | 0.820 |
| Pos + Neg5 | 76.0 | 73.0 | 73.9 | 74.5 | 0.489 | 0.748 | 0.821 |
| Pos + Neg6 | 76.0 | 74.0 | 74.8 | 75.1 | 0.501 | 0.752 | 0.826 |
| Pos + Neg7 | 75.0 | 74.0 | 73.9 | 74.2 | 0.483 | 0.743 | 0.818 |
| Pos + Neg8 | 75.0 | 74.0 | 74.0 | 74.3 | 0.485 | 0.744 | 0.824 |
| Pos + Neg9 | 75.0 | 73.0 | 73.6 | 74.1 | 0.481 | 0.743 | 0.817 |
| Pos + Neg10 | 75.0 | 74.0 | 74.5 | 74.5 | 0.490 | 0.745 | 0.823 |
| Mean | 75.2 | 73.7 | 74.1 | 74.5 | 0.490 | 0.747 | 0.822 |
| Std error | 0.52 | 0.57 | 0.40 | 0.34 | 0.007 | 0.003 | 0.003 |

**Table S3.** The performances on the independent test sets for models built on different negative subsets of *H. sapiens*.

| Data_independent | Sn% | Sp% | Pre% | Acc% | Mcc | F1-score | AUC |
| --- | --- | --- | --- | --- | --- | --- | --- |
| Pos + Neg1 | 75.0 | 80.0 | 78.8 | 77.5 | 0.551 | 0.770 | 0.858 |
| Pos + Neg2 | 75.0 | 78.0 | 77.6 | 76.8 | 0.537 | 0.765 | 0.849 |
| Pos + Neg3 | 73.0 | 77.0 | 75.8 | 74.6 | 0.493 | 0.741 | 0.829 |
| Pos + Neg4 | 71.0 | 83.0 | 80.3 | 76.8 | 0.540 | 0.754 | 0.837 |
| Pos + Neg5 | 70.0 | 88.0 | 85.7 | 79.0 | 0.590 | 0.768 | 0.861 |
| Pos + Neg6 | 70.0 | 83.0 | 80.0 | 76.1 | 0.526 | 0.744 | 0.820 |
| Pos + Neg7 | 76.0 | 81.0 | 76.8 | 71.7 | 0.443 | 0.688 | 0.828 |
| Pos + Neg8 | 75.0 | 81.0 | 80.0 | 78.3 | 0.566 | 0.776 | 0.862 |
| Pos + Neg9 | 71.0 | 81.0 | 79.0 | 76.1 | 0.524 | 0.748 | 0.804 |
| Pos + Neg10 | 68.0 | 74.0 | 72.3 | 71.0 | 0.421 | 0.702 | 0.792 |
| Mean | 72.4 | 80.6 | 78.6 | 75.8 | 0.519 | 0.746 | 0.834 |
| Std error | 2.95 | 3.88 | 3.48 | 2.64 | 0.053 | 0.029 | 0.024 |

**Table S4.** The performances on the independent test sets for models built on different negative subsets of *M. musculus*.

| Data_independent | Sn% | Sp% | Pre% | Acc% | Mcc | F1-score | AUC |
| --- | --- | --- | --- | --- | --- | --- | --- |
| Pos + Neg1 | 68.0 | 75.0 | 73.0 | 71.4 | 0.429 | 0.704 | 0.775 |
| Pos + Neg2 | 66.0 | 74.0 | 71.6 | 69.9 | 0.400 | 0.688 | 0.768 |
| Pos + Neg3 | 68.0 | 75.0 | 73.0 | 71.3 | 0.426 | 0.701 | 0.778 |
| Pos + Neg4 | 65.0 | 74.0 | 71.6 | 69.6 | 0.394 | 0.681 | 0.771 |
| Pos + Neg5 | 66.0 | 76.0 | 73.5 | 71.2 | 0.426 | 0.697 | 0.785 |
| Pos + Neg6 | 68.0 | 76.0 | 73.7 | 71.7 | 0.436 | 0.705 | 0.783 |
| Pos + Neg7 | 67.0 | 74.0 | 72.3 | 70.7 | 0.415 | 0.696 | 0.763 |
| Pos + Neg8 | 67.0 | 78.0 | 75.2 | 72.4 | 0.451 | 0.708 | 0.783 |
| Pos + Neg9 | 67.0 | 75.0 | 72.8 | 70.9 | 0.420 | 0.696 | 0.778 |
| Pos + Neg10 | 66.0 | 75.0 | 72.6 | 70.7 | 0.415 | 0.693 | 0.780 |
| mean | 66.7 | 75.2 | 72.9 | 71.0 | 0.421 | 0.697 | 0.776 |
| Std error | 0.85 | 1.19 | 1.06 | 0.82 | 0.017 | 0.008 | 0.007 |

**Table S5.** The cross validation results of KSNPF for the three species with different Ks.

| Species | K | best_ks | best_bc | Sn | Sp | Pre | Acc | Mcc | F1-score | AUC |
| --- | --- | --- | --- | --- | --- | --- | --- | --- | --- | --- |
| H. sapiens | 1 | 0.125 | 0.5 | 63.5 | 79.5 | 75.6 | 71.5 | 0.436 | 0.690 | 0.771 |
|  | 2 | 0.25 | 0.03125 | 67.5 | 77.5 | 75.0 | 72.5 | 0.452 | 0.711 | 0.784 |
|  | 3 | 1 | 32 | 73.5 | 73.0 | 73.1 | 73.3 | 0.465 | 0.733 | 0.800 |
|  | 4 | 0.5 | 16 | 73.0 | 75.5 | 74.9 | 74.3 | 0.485 | 0.739 | 0.791 |
|  | 5 | **0.25** | **4** | **73.5** | **79.5** | **78.2** | **76.5** | **0.531** | **0.758** | **0.802** |
| M. musculus | 1 | **1** | **8** | **65.5** | **64.2** | **64.7** | **64.9** | **0.298** | **0.652** | **0.702** |
|  | 2 | 0.5 | 8 | 66.5 | 60.9 | 63.0 | 63.7 | 0.275 | 0.647 | 0.691 |
|  | 3 | 0.5 | 4 | 65.1 | 62.8 | 63.6 | 63.9 | 0.279 | 0.643 | 0.691 |
|  | 4 | 4 | 2048 | 65.7 | 61.9 | 63.3 | 63.8 | 0.277 | 0.645 | 0.689 |
|  | 5 | 0.5 | 4 | 65.3 | 61.7 | 63.1 | 63.5 | 0.270 | 0.642 | 0.685 |
| A. thaliana | 1 | **0.25** | **0.125** | **57.7** | **81.0** | **75.2** | **69.4** | **0.398** | **0.653** | **0.753** |
|  | 2 | 0.5 | 2 | 54.7 | 82.9 | 76.1 | 68.8 | 0.391 | 0.636 | 0.750 |
|  | 3 | 0.25 | 0.125 | 57.9 | 80.1 | 74.4 | 69.0 | 0.390 | 0.651 | 0.749 |
|  | 4 | 0.25 | 0.0625 | 55.4 | 81.8 | 75.2 | 68.6 | 0.385 | 0.638 | 0.746 |
|  | 5 | 0.5 | 16 | 57.6 | 79.8 | 74.1 | 68.7 | 0.384 | 0.648 | 0.743 |

**Table S6.** The cross validation results of KSPSDP for the three species with different Ks.

| Species | K | best_ks | best_bc | Sn% | Sp% | Pre% | Acc% | Mcc | F1-score | AUC |
| --- | --- | --- | --- | --- | --- | --- | --- | --- | --- | --- |
| H. sapiens | 1 | 0.25 | 1 | 84.0 | 77.0 | 78.5 | 80.5 | 0.612 | 0.812 | 0.859 |
|  | 2 | 1 | 0.25 | 83.0 | 73.5 | 75.8 | 78.3 | 0.568 | 0.792 | 0.857 |
|  | 3 | 0.25 | 0.5 | 85.0 | 74.0 | 76.6 | 79.5 | 0.594 | 0.806 | 0.861 |
|  | 4 | 1 | 0.125 | 80.0 | 73.0 | 74.8 | 76.5 | 0.531 | 0.773 | 0.832 |
|  | 5 | **0.25** | **8** | **82.5** | **77.5** | **78.6** | **80.0** | **0.601** | **0.805** | **0.862** |
| M. musculus | 1 | **16** | **8192** | **73.0** | **72.6** | **72.7** | **72.8** | **0.456** | **0.728** | **0.803** |
|  | 2 | 4 | 16384 | 75.8 | 69.5 | 71.3 | 72.6 | 0.453 | 0.735 | 0.798 |
|  | 3 | 4 | 32768 | 76.0 | 67.8 | 70.3 | 71.9 | 0.440 | 0.730 | 0.792 |
|  | 4 | 0.5 | 2 | 74.5 | 69.9 | 71.2 | 72.2 | 0.444 | 0.728 | 0.792 |
|  | 5 | 0.125 | 0.5 | 74.5 | 70.1 | 71.3 | 72.3 | 0.446 | 0.729 | 0.787 |
| A. thaliana | 1 | 0.5 | 128 | 47.6 | 81.0 | 71.4 | 64.3 | 0.303 | 0.571 | 0.692 |
|  | 2 | 2 | 32768 | 51.9 | 76.6 | 68.9 | 64.3 | 0.294 | 0.592 | 0.685 |
|  | 3 | **0.0625** | **1** | **58.2** | **72.4** | **67.8** | **65.3** | **0.309** | **0.626** | **0.694** |
|  | 4 | 0.125 | 0.5 | 45.7 | 81.9 | 71.6 | 63.8 | 0.296 | 0.558 | 0.682 |
|  | 5 | 0.125 | 1 | 50.4 | 78.0 | 69.7 | 64.2 | 0.296 | 0.585 | 0.690 |
